# Comparison of Adverse Reactions Caused by Olaparib for Different Indications

**DOI:** 10.3389/fphar.2022.968163

**Published:** 2022-07-13

**Authors:** Yujing Zhou, Shengwen Zhao, Tong Wu, Han Zhang

**Affiliations:** ^1^ Department of Nuclear Medicine, Harbin Medical University Cancer Hospital, Harbin, China; ^2^ Department of Radiology, Heilongjiang Academy of Traditional Chinese Medicine, Harbin, China

**Keywords:** olaparib, pancreatic cancer, breast cancer, ovarian cancer, security

## Abstract

**Objective:** Meta-analysis of safety of Olaparib in the treatment of different indications.

**Methods:** The databases of PubMed, The Cochrane Library, EMbase, CNKI, WanFang Data and VIP were searched by computer to collect the research on the indications and the incidence of adverse reactions caused by Olaparib for different cancer types. The search time was from the establishment of the database to May 2022. After two researchers independently screened the literature, extracted the data and evaluated the bias risk included in the study, we used RevMan 5.4 software for meta-analysis.

**Results:** A total of 14 studies were included, with a total sample size of 5119 cases. By meta-analysis, the adverse reactions of Olaparib in the treatment of pancreatic cancer, breast cancer and ovarian cancer were compared. In adverse reactions of any grade, the results showed that fatigue (RR = 1.58, 95% CI [1.20–2.07], *p* = 0.001) was the most serious in the treatment of pancreatic cancer with Olaparib. Anemia (RR = 2.94, 95% CI [1.97–4.39], *p* < 0.00001), neutropenia (RR = 1.37, 95% CI [0.80–2.33], *p* = 0.25), nausea (RR = 1.93, 95% CI [1.61–2.32], *p* < 0.00001) and vomiting (RR = 1.96, 95% CI [1.59–2.41], *p* < 0.00001) were the most severe in ovarian cancer. In adverse reactions of grade 3 or above, fatigue (RR = 3.44, 95% CI [1.48–7.98], *p* = 0.004) and vomiting (RR = 1.09, 95% CI [0.42–2.81], *p* = 0.86) were the most serious adverse reactions in the treatment of breast cancer with Olaparib. Anemia (RR = 9.74, 95% CI [2.75–34.47], *p* = 0.0004), neutropenia (RR = 1.33, 95% CI [0.87–2.02], *p* = 0.19) and nausea (RR = 2.94, 95% CI [1.18–7.32], *p* = 0.02) were the most severe in ovarian cancer. In addition, the incidence of decreased white blood cell count and hepatotoxicity in the treatment of breast cancer, and the incidence of decreased platelet count, constipation and abdominal pain in the treatment of ovarian cancer were higher than those in pancreatic cancer.

**Conclusion:** Current evidence showed that the risk of adverse reactions of Olaparib in the treatment of different indications is different, and specific analysis and treatment should be carried out for different cancer types. Due to the limitation of the quantity and quality of the included studies, the above conclusions need to be verified by more high-quality studies.

## 1 Introduction

Since 2020, there have been nearly 19.3 million new cancer cases and nearly 10 million cancer patients died in the world, among which the most common types include lung cancer, breast cancer, pancreatic cancer, gastric cancer and colorectal cancer (Sung et al., 2021). The increasing incidence and mortality of cancer are threatening the lives and health of people all over the world. Although new anti-cancer drugs have been developed continuously, most of them have limited their curative effect due to their weak specificity, toxic and side effects and drug resistance. Therefore, it is urgent to find new anti-tumor drugs in clinic. Since Bryant put forward the concept of “synthetic lethality” in 2005, the potential anti-tumor effect of PARP inhibitors has been gradually revealed (Bryant et al., 2005; Farmer et al., 2005). With the development of genomics, people’s understanding of tumor molecular level is deepening, and molecular targeted drugs related to DNA repair pathway have gradually attracted people’s attention. The discovery that poly-ADP ribose polymerase (PARP) inhibitors (PARPi) selectively kill BRCA1/2 mutant cancer cells has opened up a new way for clinical treatment of tumor patients.

As one of the inhibitors of poly (ADP-ribose) polymerase (PARP), Olaparib can capture PARP at the single strand break site of DNA, prevent its repair, and produce double strand breaks that cannot be repaired accurately in tumors with defects in homologous recombination repair, such as BRCA1 or BRCA2 mutation (BRCAm), which leads to DNA damage and tumor cell death. With the continuous in-depth research of researchers, in 2018, FDA expanded the indications of Olaparib to the first-line maintenance treatment for patients with BRCA mutant ovarian cancer. Another study found that (Robson et al., 2017a; Eikesdal et al., 2021), Olaparib can significantly improve the curative effect of BRCA mutant metastatic HER2 negative breast cancer. Compared with the standard chemotherapy group, the median PFS of Olaparib group is significantly prolonged, the effective rate is significantly improved, and the side effects are less and the safety is higher. Therefore, Olaparib is the first PARPi approved for the treatment of BRCA mutant breast cancer. GOLAN (Golan et al., 2019) and other tests evaluated the role of Olaparib in patients with gBRCA1/2 mutant metastatic pancreatic cancer. Compared with placebo group, the median PFS of Olaparib group was nearly doubled, and the risk of disease progression was reduced by 47%, which opened up a new concept of pancreatic cancer maintenance treatment and promoted FDA to include BRCA mutant pancreatic cancer in the indications of Olaparib. Olaparib improves overall survival and clinical benefit, but it lacks a systematic review of its safety. In this study, the existing clinical trials of Olaparib for the above indications were searched to systematically evaluate its safety in the treatment process, in order to provide clinical evidence.

## 2 Materials and Methods

The databases of PubMed, The Cochrane Library, EMbase, CNKI, WanFang Data and VIP were searched by computer to collect the research on the incidence of adverse reactions of Olaparib in treating patients with different indications. The search time was from the establishment of the database to May 2022. The retrieval is carried out by combining subject words with free words, and is adjusted according to the characteristics of each database. At the same time, the references included in the study were searched to supplement and obtain relevant information. The search terms include Olaparib, Pancreatic Cancer, Breast Cancer, Ovarian Cancer, Safety, Adverse Reactions and so on.

Inclusion criteria: ① The types of study were RCTs and Clinical trials; The language of the literature is Chinese or English; ② Patients with pancreatic, breast and ovarian cancers treated with Olaparib were studied. ③ The outcome index is the incidence of adverse reactions of any grade and above grade 3. Exclusion criteria: ① Papers such as “Non-RCT or Clinical Trial”, “Short Case Report”, “Review”, “Review” and “Animal Experiment”; Repeated published literature; There is no report of data; The full text of the literature can not be obtained.

### 2.1 Literature Screening and Data Extraction

Two researchers independently screened the literatures, extracted the data and cross-checked them. If there is any disagreement, it will be resolved through discussion or consultation with a third party. When selecting documents, read the title first, and after excluding obviously irrelevant documents, read the abstract and full text further to determine whether to include them. If necessary, contact the original study author by email or telephone for unidentified information that is very important for this study. The contents of data extraction include: ① basic information included in the study, including the first author, published year, cancer types, types of intervention drugs, and total sample size; Number, grade and type of adverse reactions.

### 2.2 Data Extraction and Quality Assessment

The Newcastle-Ottawa scale (NOS) was used to evaluate the quality of the literature (Lazarus et al., 2019), and the quality of the included studies was evaluated according to the following eight criteria: 1) Representativeness of the exposure cohort; 2) Selection of non-exposed queues; 3) Determination of exposure method; 4) No outcome events occurred before the study began; 5) Comparability between exposed queues and non-exposed queues; 6) Evaluation of outcome events; 7) Whether the follow-up time is long enough; 8) Whether the follow-up is complete. Documents rated 7–9 are considered “high” in quality, 4–6 as “average” and 3 or less as “low”. The quality evaluation is carried out independently by two researchers and cross-checked. If there is any difference, we will ask the third researcher to help solve it.

### 2.3 Statistical Analysis

RevMan 5.4 software was used for analysis. Cochrane χ^2^ test was used to evaluate the heterogeneity, and *I* ^*2*^ was used to express the heterogeneity. When *p* > 0.1 and *I* ^*2*^ < 50%, it shows that there is no statistical heterogeneity in each RCTs, and fixed effect model is used; On the contrary, on the premise of excluding clinical heterogeneity, the random effect model is adopted. Relative risk (RR) or odds ratio (OR) was used to describe the data of the two-class variables. Weighted mean difference (WMD) was used to describe the continuity variables. The effect variables were expressed by 95% confidence interval (95% CI). Sensitivity analysis was made for each included literature, and the bias of the included literature was discussed.

## 3 Results

### 3.1 Literature Search and Screening

**FIGURE 1:**
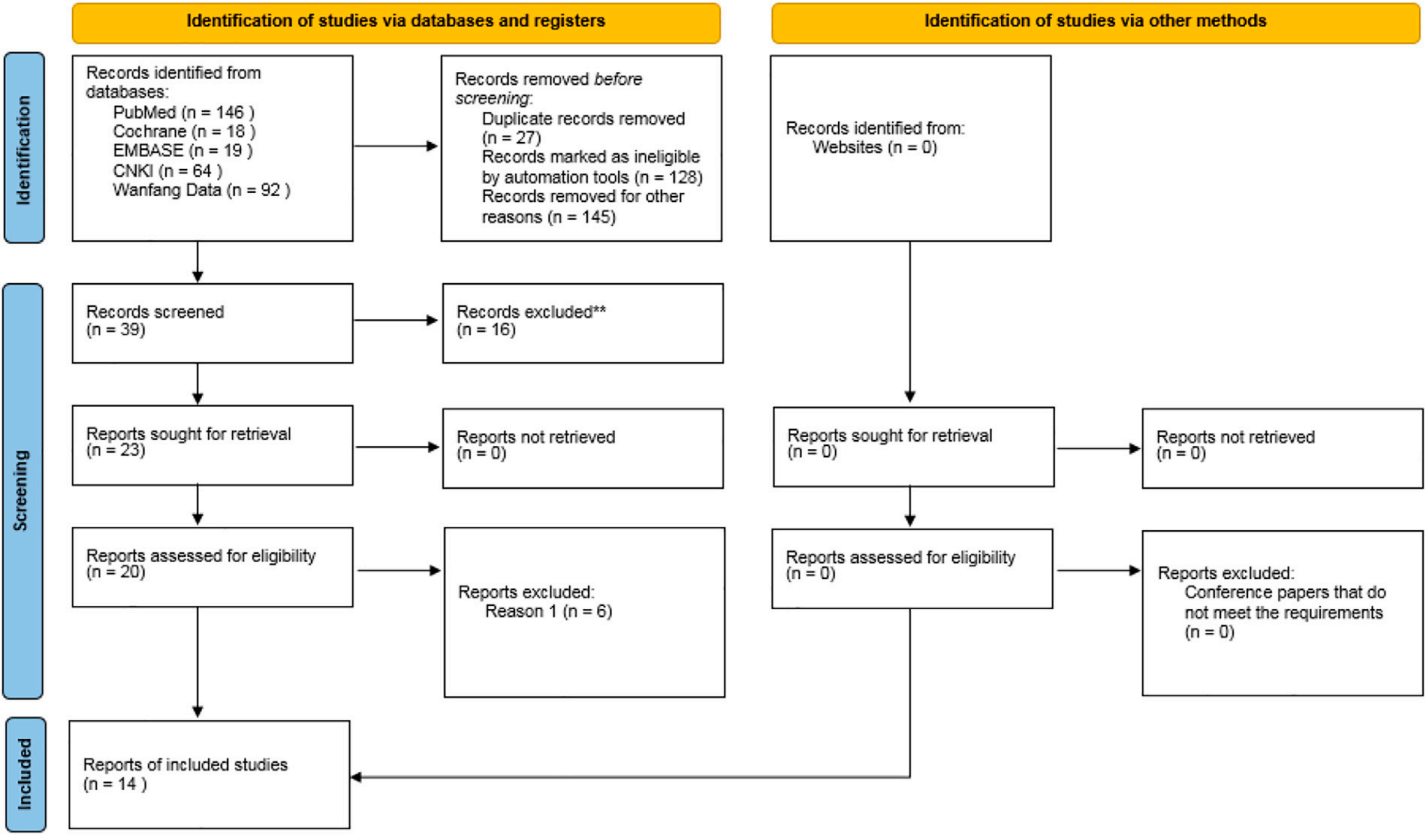
Flow chart of literature screening process and results.

A total of 339 related literatures were obtained through database search, 27 duplicate literatures were eliminated, 128 inconsistent literatures were eliminated by reading titles and abstracts, and 145 literatures were eliminated by reading full texts. After strict hierarchical screening, 14 RCTs (Ledermann et al., 2012; Bendell et al., 2015; Oza et al., 2015; Robson et al., 2017b; Moore et al., 2018; Golan et al., 2019; Hammel et al., 2019; Ray-Coquard et al., 2019; Robson et al., 2019; Im et al., 2020; Penson et al., 2020; Fasching et al., 2021; Poveda et al., 2021; Tutt et al., 2021) were finally included, including 5119 patients, including 3039 cases in Olaparib group and 2080 cases in non-Olaparib group, as shown in Figure 1. The basic characteristics and bias risk assessment results of the 14 studies included are shown in Table 1.

**TABLE 1:** Basic characteristics of included studies (Ledermann et al., 2012; Bendell et al., 2015; Oza et al., 2015; Robson et al., 2017b; Moore et al., 2018; Golan et al., 2019; Hammel et al., 2019; Ray-Coquard et al., 2019; Robson et al., 2019; Im et al., 2020; Penson et al., 2020; Fasching et al., 2021; Poveda et al., 2021; Tutt et al., 2021).

First author	Year	Phase	Cancer	ID of Study	Protocol of olaparib	Protocol of Non-olaparib	No. of olaparib	No. of Non-olaparib	Quality
Bendell et al. (2015)	2015	I	PC	NCT00515866	Olaparib + Gemcitabine	Gemcitabine	15	7	7
Hammel et al. (2019)	2019	III	PC	NCT02184195	Olaparib	Placebo	89	58	7
Golan et al. (2019)	2019	NA	PC	NA	Olaparib	Placebo	91	60	8
Tutt et al. (2021)	2021	III	BC	NCT02032823	Olaparib	Placebo	921	915	8
Robson et al. (2017b)	2017	III	BC	NCT02000622	Olaparib	Standard-Therapy	205	97	8
Im et al. (2020)	2020	III	BC	NCT02000622	Olaparib	Chemotherapy TPC	59	28	7
Robson et al. (2019)	2019	III	BC	NCT02000622	Olaparib	TPC	205	97	7
Fasching et al. (2021)	2020	III	BC	NCT02789332	Olaparib + paclitaxel	carboplatinum + paclitaxel	69	37	7
Penson et al. (2020)	2020	III	OC	NCT00628251	Olaparib	chemotherapy	178	76	7
Ledermann et al. (2012)	2012	II	OC	NCT00753545	Olaparib	placebo	136	128	8
Moore et al. (2018)	2018	III	OC	NCT01844986	Olaparib	chemotherapy	260	130	8
Ray-Coquard et al. (2019)	2019	III	OC	NCT02477644	Olaparib	chemotherapy	535	267	8
Oza et al. (2015)	2014	II	OC	NCT01081951	Olaparib	chemotherapy	81	81	8
Poveda et al. (2021)	2021	III	OC	NCT01874353	Olaparib	placebo	195	99	7

PC, pancreatic cancer; BC, breast cancer; OC, ovarian cancer; TPC, treatment of physician’s choice; NA, not available.

### 3.2 Meta-Analysis Results

#### 3.2.1 Major Adverse Reactions

##### 3.2.1.1 Meta-Analysis Results of Fatigue of Any Level and Above Caused by Olaparib

**FIGURE 2:**
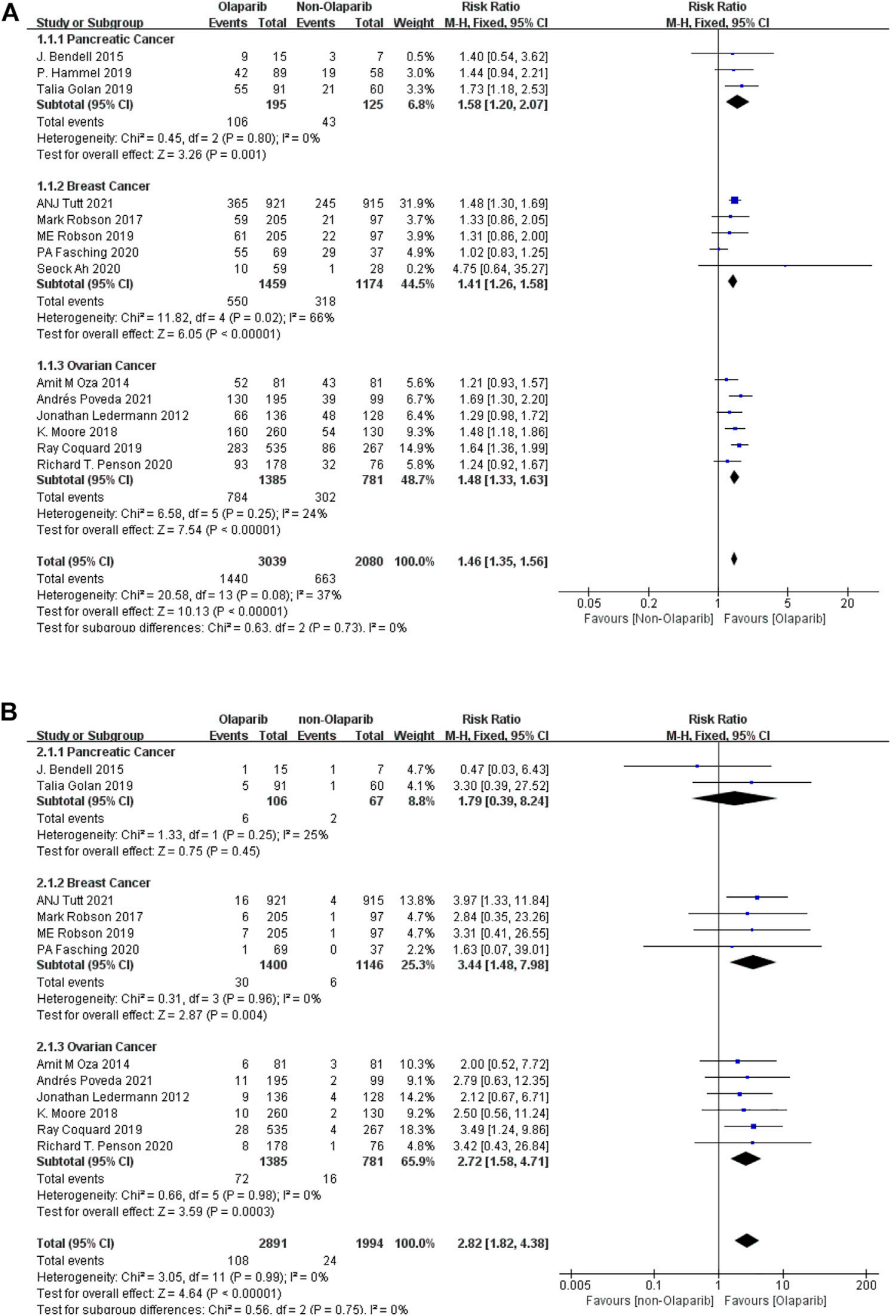
**(A)** Meta-analysis of any-grade adverse reactions of fatigue caused by Olaparib for different indications. **(B)** Meta-analysis of grade 3 or above adverse reactions of fatigue caused by Olaparib for different indications.

Meta-analysis according to cancer types showed that: ① Among the adverse reactions of any grade in 14 studies, the risk of fatality was the highest in pancreatic cancer (RR = 1.58, 95% CI [1.20–2.07], *p* = 0.001), followed by ovarian cancer (RR = 1.48, 95% CI [1.33–1.63], *p* < 0.00001), and relatively low in breast cancer (RR = 1.41, 95% CI [1.26–1.58], *p* < 0.00001). The results were statistically significant (*p* < 0.05), as shown in Figure 2A. ② Among the 13 studies, the risk of severe adverse reactions above grade 3 was the highest in breast cancer (RR = 3.44, 95% CI [1.48–7.98], *p* = 0.004), followed by ovarian cancer (RR = 2.72, 95% CI [1.58–4.71], *p* = 0.0003), and relatively low in pancreatic cancer (RR = 1.79, 95% CI [0.39–8.24], *p* = 0.45). Some results were statistically significant (*p* < 0.05), as shown in Figure 2B. The results suggest that in the routine grade of adverse reactions, Olaparib is more likely to cause false when treating pancreatic cancer, while in the severe adverse reactions, more attention should be paid to the degree of false when treating breast cancer.

##### 3.2.1.2 Meta-Analysis Results of Anemia of Any Level and Above Caused by Olaparib

**FIGURE 3:**
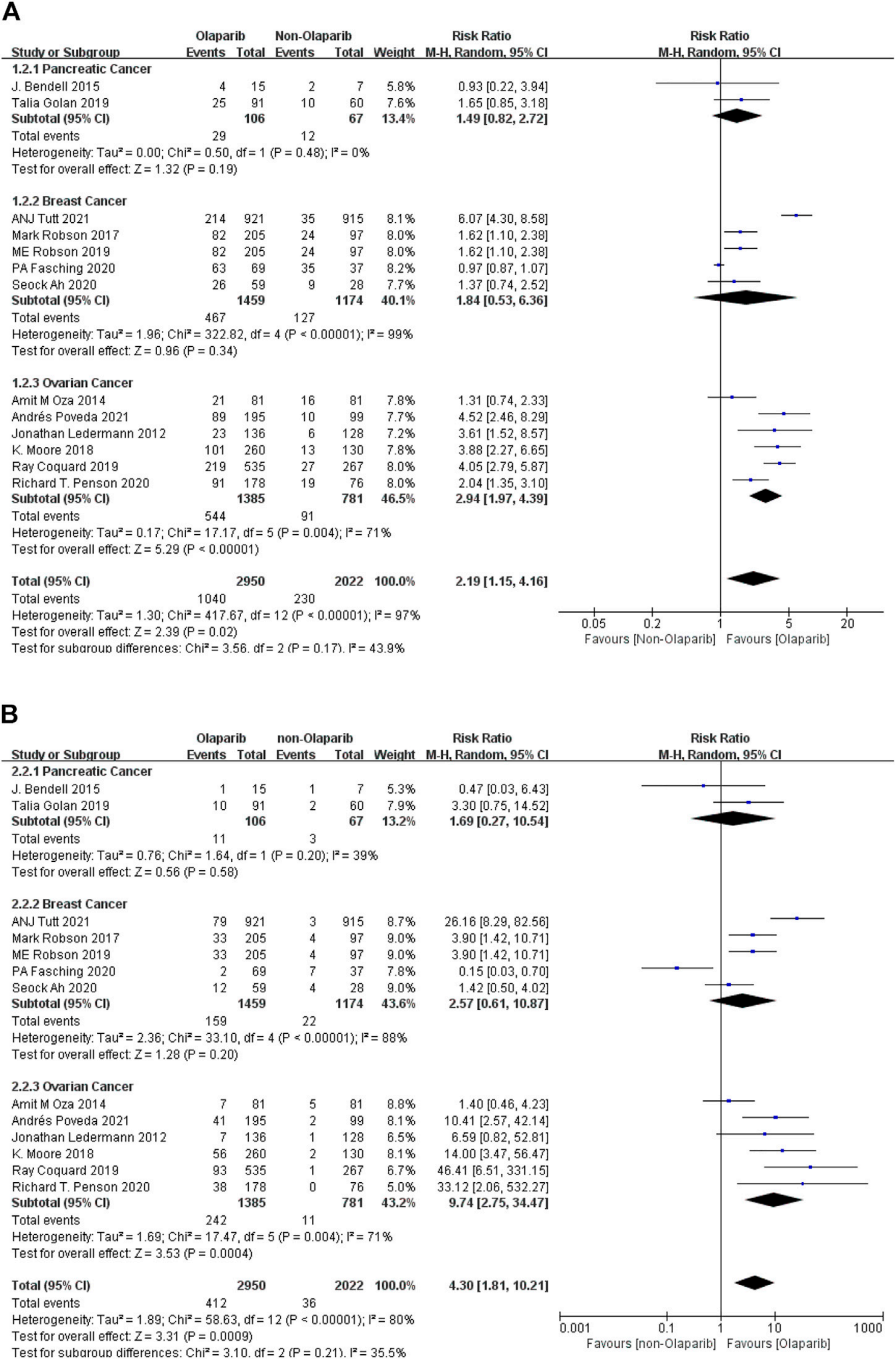
**(A)** Meta-analysis of any-grade adverse reactions of anemia caused by Olaparib for different indications. **(B)** Meta-analysis of grade 3 or above adverse reactions of anemia caused by Olaparib for different indications.

The results of meta-analysis were as follows: ① Among the adverse reactions of any grade in 14 studies, anemia had the highest risk in ovarian cancer (RR = 2.94, 95% CI [1.97–4.39], *p* < 0.00001), followed by breast cancer (RR = 1.84, 95% CI [0.53–6.36], *p* < 0.00001), and relatively low risk in pancreatic cancer (RR = 1.49, 95% CI [0.82–2.72], *p* < 0.00001). Some results were statistically significant (*p* < 0.05), as shown in Figure 3A. ② Among the 13 studies, anemia had the highest risk in ovarian cancer (RR = 9.74, 95% CI [2.75–34.47], *p* = 0.0004), followed by breast cancer (RR = 2.57, 95% CI [0.61–10.87], *p* = 0.20), and relatively low risk in pancreatic cancer (RR = 1.69, 95% CI [0.27–10.54], *p* = 0.58). Some results were statistically significant (*p* < 0.05), as shown in Figure 3B. The results suggest that in the clinical application of Olaparib, compared with breast cancer, Olaparib is more likely to cause anemia in the treatment of ovarian cancer.

##### 3.2.1.3 Meta-Analysis Results of Nausea of Any Grade and Above Caused by Olaparib

**FIGURE 4:**
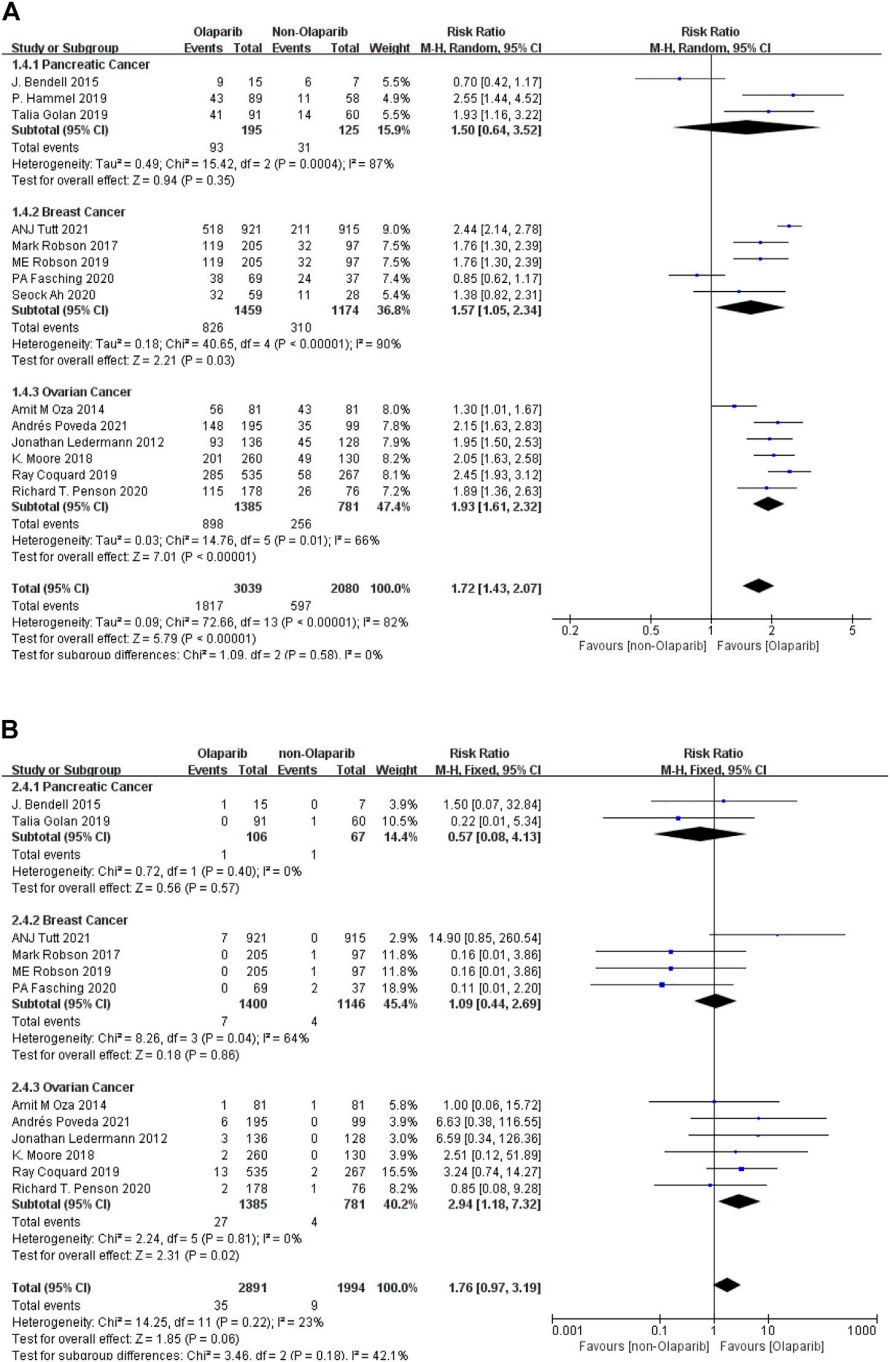
**(A)** Meta-analysis of any-grade adverse reactions of nausea caused by Olaparib for different indications. **(B)** Meta-analysis of grade 3 or above adverse reactions of nausea caused by Olaparib for different indications.

The results of meta-analysis were as follows: ① Among the 14 studies, nausea had the highest risk in ovarian cancer (RR = 1.93, 95% CI [1.61–2.32], *p* < 0.00001), followed by breast cancer (RR = 1.57, 95% CI [1.05–2.34], *p* = 0.03), and relatively low risk in pancreatic cancer (RR = 1.50, 95% CI [0.64–3.52], *p* = 0.35). Some results were statistically significant (*p* < 0.05), as shown in Figure 4A. ② Among the adverse reactions above grade 3 in 13 studies, nausea has the highest risk in ovarian cancer (RR = 2.94, 95%CI [1.18–7.32], *p* = 0.02), followed by breast cancer (RR = 1.09, 95% CI [0.44–2.69], *p* = 0.86), and relatively low risk in pancreatic cancer (RR = 0.57, 95% CI [0.08–4.13], *p* = 0.57). Some results are statistically significant (*p* < 0.05), as shown in Figure 4B. The results suggest that in the clinical application of Olaparib, compared with the treatment of breast cancer, Olaparib is more likely to cause nausea in the treatment of ovarian cancer.

### 3.3 Secondary Adverse Reaction

Subgroup analysis of secondary adverse reactions was conducted according to different types of cancer, and the results of meta-analysis were as follows: ① Based on existing clinical evidence, decreased white blood cell count, hepatotoxicity (increased AST and ALT) only occurred in breast cancer, and only decreased AST (RR = 0.62, 95% CI [0.46–0.82], *p* = 0.0009) and ALT (RR = 0.74, 95% CI [0.58–0.94], *p* = 0.01) the results were statistically significant (*p* < 0.05). ② Thrombocytopenia mainly occurred in ovarian cancer, and there was statistical difference in thrombocytopenia at any grade (RR = 2.06, 95% CI [1.46–2.92], *p* < 0.0001) (*p* < 0.05). ③ Neutrophilic granulocyte reduction of any grade and above grade 3 had the highest risk of ovarian cancer (RR = 1.37, 95% CI [0.80–2.33], *p* = 0.25) and (RR = 1.33, 95% CI [0.87–2.02], *p* = 0.19), respectively. ④ All levels of depression had the highest risk in ovarian cancer (RR = 1.96, 95% CI [1.59–2.41], *p* < 0.00001), followed by breast cancer (RR = 1.78, 95% CI [1.14–2.77], *p* = 0.57). The lowest risk was found in pancreatic cancer (RR = 1.22, 95% CI [0.61–2.47], *p* = 0.57). The risk of tertiary depression was the highest in breast cancer (RR = 1.09, 95% CI [0.42–2.81], *p* = 0.86). ⑤ Cases of diarrhea and Abdominal pain are only in pancreatic cancer and ovarian cancer, and the risk of both are high in pancreatic cancer (RR = 2.49, 95% CI [1.54–4.02], *p* = 0.0002) and (RR = 0.99, 95% CI [0.76–1.30], *p* = 0.96; ⑥ Appetite decreased is mainly found in pancreatic cancer, with the highest incidence (RR = 2.29, 95% CI [1.39–3.77], *p* = 0.001), and second in breast cancer (RR = 1.92, 95% CI [1.51–2.45], *p* < 0.00001). The incidence was lowest in ovarian cancer (RR = 1.59, 95% CI [1.20–2.12], *p* = 0.001) as shown in Table 2.

**TABLE 2:** Meta-analysis of secondary adverse reactions of Olaparib for different indications.

	Pancreatic cancer		Breast cancer		Ovarian cancer	
	Any-Grade ADR	Grade 3 or Above ADR	Any-Grade ADR	Grade 3 or Above ADR	Any-Grade ADR	Grade 3 or Above ADR
WBC count decreased	NA	NA	1.11 [0.53–2.34], *p* = 0.77	0.78 [0.18–3.50], *p* = 0.77	NA	NA
Platelet count decreased	NA	NA	NA	NA	2.06 [1.46–2.92], *p* < 0.0001	1.36 [0.68–2.73], *p* = 0.39
Neutrophil count decreased	0.78 [0.25–2.37], *p* = 0.66	0.78 [0.25–2.37], *p* = 0.66	0.79 [0.42–1.48], *p* = 0.46	0.67 [0.26–1.73], *p* = 0.40	1.37 [0.80–2.33], *p* = 0.25	1.33 [0.87–2.02], *p* = 0.19
Vomiting	1.22 [0.61–2.47], *p* = 0.57	0.96 [0.13–7.17], *p* = 0.97	1.78 [1.14–2.77], *p* = 0.57	1.09 [0.42–2.81], *p* = 0.86	1.96 [1.59–2.41], *p* < 0.00001	1.02 [0.49–2.16], *p* = 0.95
Constipation	2.49 [1.54–4.02], *p* = 0.0002	NA	NA	NA	1.03 [0.85–1.26], *p* = 0.74	NA
Appetite decreased	2.29 [1.39–3.77], *p* = 0.001	NA	1.92 [1.51–2.45], *p* < 0.00001	NA	1.59 [1.20–2.12], *p* = 0.001	NA
Abdominal pain	0.99 [0.76–1.30], *p* = 0.96	0.60 [0.11–3.19], *p* = 0.55	NA	NA	1.04 [0.87–1.23], *p* = 0.67	0.83 [0.43–1.60], *p* = 0.57
AST	NA	NA	0.62 [0.46–0.82], *p* = 0.0009	2.83 [0.74–10.85], *p* = 0.13	NA	NA
ALT	NA	NA	0.74 [0.58–0.94], *p* = 0.01	0.78 [0.25–2.38], *p* = 0.66	NA	NA

WBC, white blood cell; ADR, adverse reactions; AST, aspartate aminotransferase; ALT, alanine aminotransferase; NA, not available.

### 3.4 Publication Bias

In addition to meta-analysis and comparison of data indicators of adverse reactions of any grade and those above grade 3, an inverted funnel plot was also drawn for the included studies (due to the large number of outcome indicators in this study, only language description was made). The results showed that the funnel plot of outcome indicators could be symmetrical in any level of adverse reactions. In the adverse reactions above level 3, only the funnel diagram of anemia and Neutrophil indicators was asymmetric, suggesting a certain publication bias. There may be some factors, such as lack of research design and relatively poor methods, leading to a small bias.

### 3.5 Sensitivity Analysis

Sensitivity analysis was carried out by excluding individual studies one by one, and the results showed little change compared with the total adverse reaction rate, which indicated that the results of this study were stable.

## 4 Discussion

Many studies have confirmed that maintenance treatment with poly ADP-ribose polymerase (PARP) inhibitors can significantly prolong the survival time of cancer patients such as breast cancer and ovarian cancer, and improve the survival benefits of patients. With the wide application of PARP inhibitors, drug-related adverse reactions have attracted much attention. The adverse reactions of PARP inhibitors include blood toxicity, digestive tract toxicity, etc. As one of PARPi, Olaparib also has corresponding adverse reactions.

Hematological adverse reactions of PARP inhibitors include anemia, thrombocytopenia, neutropenia and so on. Such adverse reactions mainly occur in the first 3 months of treatment, and sometimes it is necessary to interrupt or reduce the dose of PARP inhibitor for recovery. The whole blood cell count should be monitored weekly in the first month of PARP inhibitor treatment, monthly in the first year of treatment and regularly thereafter. In case of suspension of treatment due to grade 3 or 4 hematological adverse reactions, consider monitoring the whole blood cell count every week after resuming medication until it returns to normal level (Madariaga et al., 2020). Anemia is the most common blood adverse reaction. Studies have shown that (Farrés et al., 2013; Hopkins et al., 2019), PARP inhibitors can target PARP1, PARP2, PARP3 and PARP13. PARP1 regulates cell differentiation in bone marrow or blood system, and PARP2 plays a role in regulating erythropoiesis. The incidence of Olaparib anemia is 21 ∼ 46%, the incidence of grade 3 ∼ 4 anemia is about 5.1 ∼ 22%, the incidence of thrombocytopenia is 16 ∼ 18%, the incidence of grade 3 ∼ 4 is less than 1%, the incidence of neutropenia is 16 ∼ 23%, and the incidence of grade 3 ∼ 4 is 3.7 ∼ 9%. Non-hematological adverse reactions of PARP inhibitors include digestive tract toxicity and hepatotoxicity. Such adverse reactions generally occur in the first 4 ∼ 8 weeks of treatment, which is relatively short. Most patients can pass symptom management without suspending administration or reducing dosage. Among them, nausea and vomiting are the most common digestive system adverse reactions of PARP inhibitors. The incidence of nausea is relatively high, and it mainly occurs in the early stage. The overall incidence of Olaparib is 44 ∼ 73%, and the incidence of grade 3 ∼ 4 is 1 ∼ 2%. Fatigue is also a common adverse reaction of PARP inhibitors. The overall incidence of fatigue in Olaparib is 42 ∼ 63%, and the incidence of grade 3 ∼ 4 is 4.1 ∼ 7.4%.

In recent years, FDA has expanded the indications of olapalil, and used it for the treatment of breast cancer and ovarian cancer before and after. Another clinical trial shows that it also has a good clinical benefit rate for pancreatic cancer. With the wide clinical use of Olaparib, its adverse reactions have attracted much attention. The innovation of this study is to evaluate the risk of adverse reactions in the treatment of pancreatic cancer, breast cancer and ovarian cancer. Subgroup analysis was carried out according to cancer types to observe which adverse reactions in different cancer types have higher risk and deserve clinical attention. This study included 14 clinical trials and RCTs, with a total of 5119 patients. Among them, there are 3 pancreatic cancer diseases, 5 breast cancer diseases and 6 ovarian cancer diseases. Subgroup analysis showed that there were significant differences in the incidence of adverse reactions among patients with different cancer types. Based on the existing clinical evidence, for the main adverse reactions, fatigue of any level has the highest risk in pancreatic cancer, followed by ovarian cancer and relatively low risk in breast cancer; Fatigue above grade three has the highest risk in breast cancer, followed by ovarian cancer and relatively low risk in pancreatic cancer. Anemia and nausea of any grade and above are at the highest risk in ovarian cancer, followed by breast cancer and relatively low risk in pancreatic cancer. The above results suggest that in clinical use, Olaparib is more prone to fatigue adverse reactions in the treatment of pancreatic cancer patients, while anemia and nausea adverse reactions should be paid more attention to in the treatment of breast cancer and ovarian cancer patients, and similar symptoms should be treated correctly in time. For minor adverse reactions, when Olaparib treats breast cancer patients, it should pay more attention to the decrease of white blood cell count and hepatotoxicity; Attention should be paid to the changes of platelet count and neutrophil count in the treatment of ovarian cancer patients. Constipation, abdominal pain and loss of appetite often occur when treating pancreatic cancer patients.

The quality assessment of this study is shown in Table 1, the 14 included literatures were all high-quality literatures, of which 7 literatures scored 7 points and 7 literatures scored 8 points.

There are also some limitations in this study. Due to the short time to market of Olaparib and limited clinical data for some indications, our study is essentially a meta-analysis based on available data in published literature. Although we have made great efforts to collect as much information as possible, the number of studies included has limited our further analysis to some extent and affected our results. In the later period, the corresponding clinical trial data will be continuously supplemented to consolidate the results of this study, expecting to provide a more reliable basis for clinical medication.

In conclusion, according to the current evidence, the risk of adverse reactions of Olaparib in the treatment of different indications is different, and specific analysis and treatment should be carried out for different cancer types.

## Data Availability

The original contributions presented in the study are included in the article/supplementary material, further inquiries can be directed to the corresponding author.
